# PP2A-B55alpha controls keratinocyte adhesion through dephosphorylation of the Desmoplakin C-terminus

**DOI:** 10.1038/s41598-023-37874-8

**Published:** 2023-08-05

**Authors:** Abbey L. Perl, Jennifer L. Koetsier, Kathleen J. Green

**Affiliations:** 1 Department of Pathology, Feinberg School of Medicine, Northwestern University, 303 E Chicago Ave., Chicago, IL 60611 USA; 2 Department of Dermatology, Feinberg School of Medicine, Northwestern University, Chicago, IL USA; 3 Robert H. Lurie Comprehensive Cancer Center, Northwestern University, Chicago, IL USA

**Keywords:** Phosphorylation, Desmosomes, Intermediate filaments

## Abstract

Critical for the maintenance of epidermal integrity and function are attachments between intermediate filaments (IF) and intercellular junctions called desmosomes. The desmosomal cytoplasmic plaque protein desmoplakin (DP) is essential for anchoring IF to the junction. DP-IF interactions are regulated by a phospho-regulatory motif within the DP C-terminus controlling keratinocyte intercellular adhesion. Here we identify the protein phosphatase 2A (PP2A)-B55α holoenzyme as the major serine/threonine phosphatase regulating DP’s C-terminus and consequent intercellular adhesion. Using a combination of chemical and genetic approaches, we show that the PP2A-B55α holoenzyme interacts with DP at intercellular membranes in 2D- and 3D- epidermal models and human skin samples. Our experiments demonstrate that PP2A-B55α regulates the phosphorylation status of junctional DP and is required for maintaining strong desmosome-mediated intercellular adhesion. These data identify PP2A-B55α as part of a regulatory module capable of tuning intercellular adhesion strength and a candidate disease target in desmosome-related disorders of the skin and heart.

## Introduction

Cytoskeletal-associated intercellular adhesion junctions are essential for maintaining the stability of multi-cellular tissues and providing cells with the structural integrity to withstand the changing mechanical environment of the tissue. This is particularly important in tissues experiencing high external or internal levels of mechanical stress such as the stratified epidermis or heart. Particularly important for these tissues are desmosomes, intercellular junctions that integrate chemical and mechanical stimuli to allow for the dynamic regulation of the cortical cytoskeleton^1,2^.

The desmosome comprises transmembrane cadherins from two families, desmogleins (Dsg) and desmocollins (Dsc), two plaque armadillo proteins, plakophilin (Pkp) and plakoglobin (PG), and an intermediate filament (IF) cytoskeletal linker protein, desmoplakin (DP). By tethering the IF cytoskeleton to the plasma membrane, DP strengthens adhesion and distributes forces throughout tissues^1,2^. Unlike the Dsg, Dsc, and Pkp families, which are regulated by isoform expression at specific differentiated layers in the stratified epidermis, DP is the only desmosome plakin protein and is therefore ubiquitously expressed in all desmosome-forming cells^3^. The essential nature of DP is best highlighted by the embryonic lethal phenotype of DP null mice, and severe defects exhibited in high-tension tissues including the skin and heart of tetraploid rescue and epidermal specific knockout mice^4–6^. In addition, mutations in the *DSP* gene result in a range of disorders from lethal acantholytic epidermolysis bullosa (LAEB) to striate palmar plantar keratoderma (SPPK) as well as arrhythmogenic cardiomyopathy (AC) and cardiocutaneous Carvajal syndrome^7–13^.

**Figure 1:**
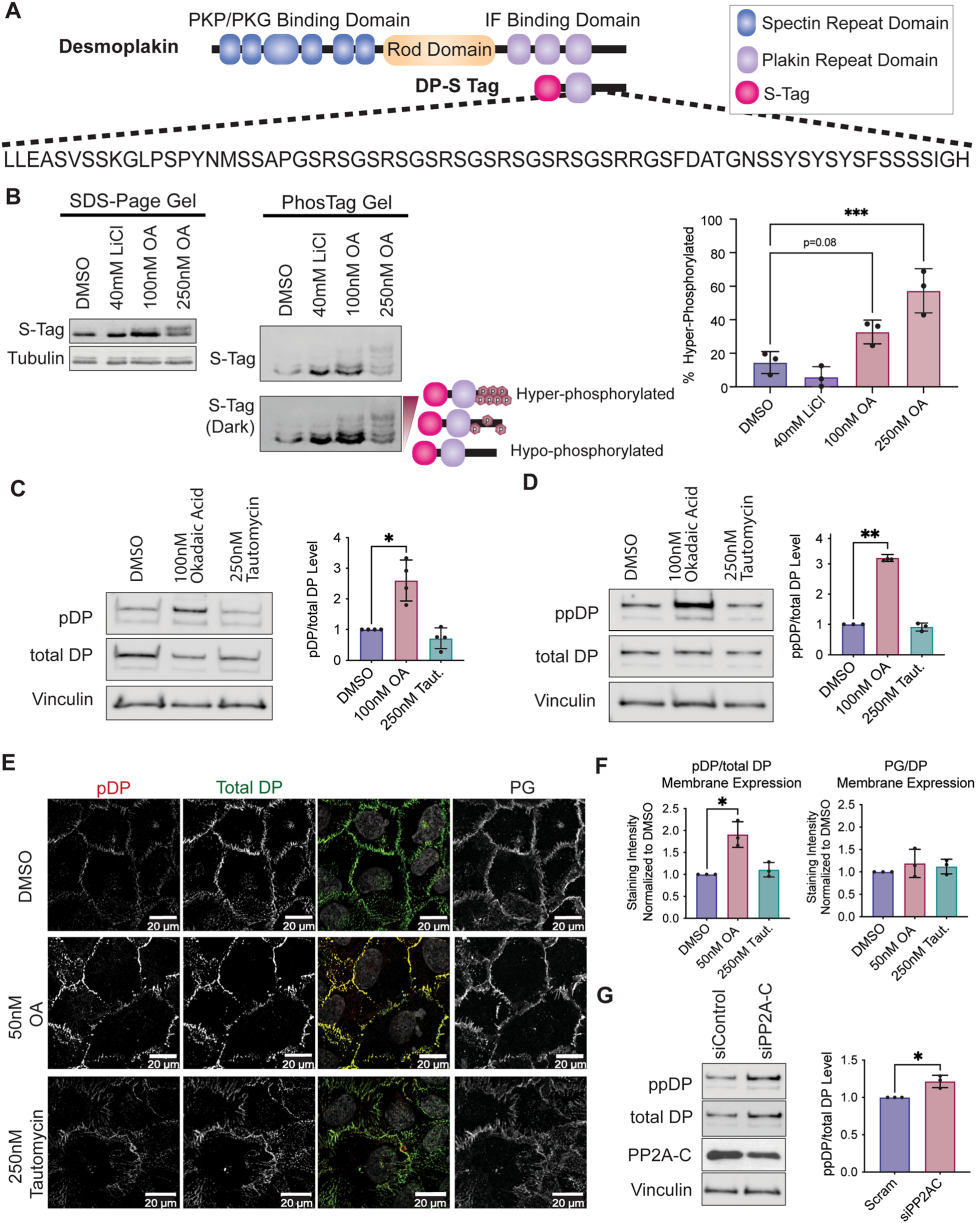
PP2A is a phosphatase for the C-terminal domain of DP. (**A**) Schematic of DP structural domains and the DP-S-Tag construct containing only residues 2628–2871. Highlighted below is the 68 residue-long “GSR” repeat phospho-regulatory motif downstream of the IF binding site capable of regulating DP-IF interactions. (**B**) A 15% acrylamide gel without (left) and with (right) Phos-Tag molecule capable of separating protein by its number of phosphate groups. Protein lysates are from SCC9 cells transfected with the DP-S Tag C-terminus and treated with either LiCl or OA for 3 h. Phos-Tag gel was analyzed using an anti-S-Tag antibody to visualize the total DP-S-Tag expression. Quantification of the % Phosphorylated DP-Stag is on right. Percent hyper-phosphorylated was calculated by (phosphorylated signal/total signal)*100 to get a final percentage. (**C**–**F**) SCC9s were treated with inhibitors preferential for either PP2A (50 and 100 nM OA) or PP1 (250 nM Tautomycin) for 3 h. Western blot analysis of S2849 phosphorylated DP (pDP) (**C**) and the dual S2845/S2849 phosphorylated DP (ppDP) (**D**). (**E**) Immunofluorescence staining of S2849 phosphorylated DP (pDP) in drug treated SCC9’s. (**F**) Staining intensity from [E] was quantified at the membrane using PG stain as a mask. (**G**) Amount of the dual S2845/S2849 phosphorylated DP (ppDP) was analyzed in total SCC9 cells transfected with siRNA targeting the PP2A-C subunit. Statistical analyses were performed on normalized data using One-way ANOVA with multiple comparisons (**B**–**F**) or a student t-test (**G**). * < 0.05; ** < 0.01; *** < 0.001.

Notably, DP is regulated in part by the processive phosphorylation of a glycine-serine-arginine repeat at its C-terminus directly downstream of the DP-IF binding site (Fig. 1A)^14,15^. Previous work from our group and others have characterized the hypo-phosphorylated form of DP as having increased IF binding affinity that impacts desmosome formation, dynamics, and function^14,16–18^. Specifically, expression of constitutively hypo-phosphorylated DP mutants increased adhesion strength and tissue stiffness, resulting in epidermal cell sheets able to withstand higher mechanical stress^17–19^. The phosphorylation of DP’s phospho-regulatory motif was previously identified to be triggered by the coordinated activity of the glycogen synthase kinase 3β (GSK3β) and the protein arginine methyltransferase 1 (PRMT-1)^14^. Despite the importance of the hypo-phosphorylated form of DP in strengthening IF binding, the phosphatase responsible for dephosphorylating DP remained unknown.

This study identifies the protein phosphatase 2A (PP2A)-B55α holoenzyme as the phosphatase responsible for de-phosphorylating DP’s C-terminus. Using a combination of chemical and genetic approaches, we show that inhibition of the PP2A-B55α holoenzyme induces an increase in DP phosphorylation at sites of intercellular junctions. Additionally, we find that the PP2A regulatory subunit B55α is associated with DP at intercellular membranes where both DP and B55α localization is dependent on the other’s expression. Lastly, keratinocyte intercellular adhesion assays showed that loss of the PP2A-B55α holoenzyme diminishes intercellular adhesion strength mediated by the DP C-terminus phospho-motif. Collectively, these data reveal the existence of a previously unrecognized regulatory module capable of fine-tuning desmosome-dependent adhesion. We propose that this module provides a mechanism for rapid adaptations necessary for maintaining a functional barrier in the dynamic mechanical environment of the epidermis.

## RESULTS

### PP2A regulates the phosphorylation of DP’s C-terminus

The importance of hypo-phosphorylated DP for stabilizing the DP-IF interaction to strengthen desmosome-mediated adhesion suggests the existence of a phosphatase that dephosphorylates DP in a regulated matter. To determine if serine/threonine protein phosphatases are involved in this process, keratinocytes were treated with the pan-serine/threonine phosphatase inhibitor Okadaic Acid (OA). To afford comparisons with previous data identifying GSK3β and PRMT-1 as regulating the phosphorylation of DP’s C-terminus, we initially chose to use the squamous cell carcinoma cell line SCC9^14^. SCC9 cells were transfected with a 32 kDa truncated form of DP (DP-S-Tag, Fig. 1A) containing only its C-terminal domain including residues 2628–2871^14^. DP-S-Tag expressing cells were treated with 100 nM OA, 250 nM OA, or DMSO vehicle control for 3 h. As an internal control, cells were treated with 40 mM of lithium chloride (LiCl), a GSK3β inhibitor known to induce a hypo-phosphorylated DP C-terminus^14^. Cell lysates were collected and run on gels containing a Phos-tag peptide capable of separating proteins based on the number of phosphate groups present^20^. The Phos-tag gel showed that LiCl treatment induced an increase of hypo-phosphorylated DP, as evident by an accumulation of the lower-most DP-S-Tag band (Fig. 1B). Treatment with 100 nM OA increased the intensity of the second band from the bottom representing an increase in partially phosphorylated DP, while 250 nM OA treatment resulted in an accumulation of upper S-Tag bands representing hyper-phosphorylated DP. Quantification of the Phos-Tag gel was performed by determining the total DP S-tag signal and the hyper-phosphorylated DP Stag signal, and percent hyper-phosphorylated was calculated by (hyper-phosphorylated signal/total signal)*100 to get a final percentage.

To identify the phosphatase involved in dephosphorylating DP’s C-terminus, we employed chemical inhibitors targeting the two major serine/threonine protein phosphatases that together account for roughly 90% of all serine/threonine phosphatase activity in keratinocytes, PP2A and PP1. While phosphatase inhibitors exhibit cross-reactivity in their substrates, their preferential selectivity allows for differential inhibition of PP2A and PP1. OA preferentially inhibits PP2A (IC50 =  ~ 0.1 nM) with an affinity 10 × greater than its next major target PP1, (IC50 =  ~ 15 nM) while Tautomycin preferentially inhibits PP1 (PP1 IC50 =  ~ 0.2 nM, PP2A IC50 =  ~ 1 nM)^21^. Therefore, SCC9 cells were treated with either 100 nM OA, 250 nM Tautomycin, or DMSO vehicle control for 3 h. A phospho-antibody specific to the S2849 site on DP’s C-terminus was used as a readout for DP C-terminus phosphorylation. OA, but not Tautomycin, treatment increased the levels of phosphorylated DP in total cell lysates (Fig. 1C). Moreover, PP2A inhibition induced an increase in a dual-phosphorylated form of DP as measured using an antibody targeting residues S2845 and S2849 within the DP C-terminus (Fig. 1D), consistent with a role for PP2A in regulating multiple residues within the C-terminal phospho-serine cascade.

To determine if PP2A inhibition affects DP phosphorylation at cell–cell junctions, immunofluorescence staining of phosphorylated and total DP in SCC9s treated with drug for 3 h was performed. As the higher dose of 100 nM OA detrimentally affected cell morphology and cell contacts, a lower dose, 50 nM OA, that had no visible toxicity or morphological affects was used alongside 250 nM Tautomycin. Even with 50 nM OA the amount of phosphorylated DP at cell–cell membranes increased almost twofold, while 250 nM Tautomycin had no detectable impact on the level of phosphorylated DP at the cell membrane (Fig. 1E–F). Moreover, there was no detectable change in the total amount of membrane-associated DP, suggesting changes detected were specific to DP phosphorylation. This is consistent with data generated in the spontaneously immortalized, non-transformed keratinocyte cell line HaCaT (Supplemental Fig. 1A) as well as in primary neonatal human epidermal keratinocyte (NHEK) cells (Supplemental Fig. 1B). To further confirm the effects on DP’s phosphorylation was specific to PP2A activity, SCC9 cells were transfected with siRNA targeting PP2A’s catalytic subunit (PP2A-C) or a Scramble control sequence and grown at confluency for 2 days. Similar to OA treatment, knockdown of PP2A-C increased the level of dual-phosphorylated DP (Fig. 1G). This remained consistent in HaCaT cells where PP2A-C knockdown increased phospho-S2849 DP levels at cell membranes (Supplemental Fig. 2). Based on these initial data suggesting PP2A, and not PP1, is responsible for regulating DP’s C-terminus, we focused on further elucidating the PP2A-DP relationship.

**Figure 2:**
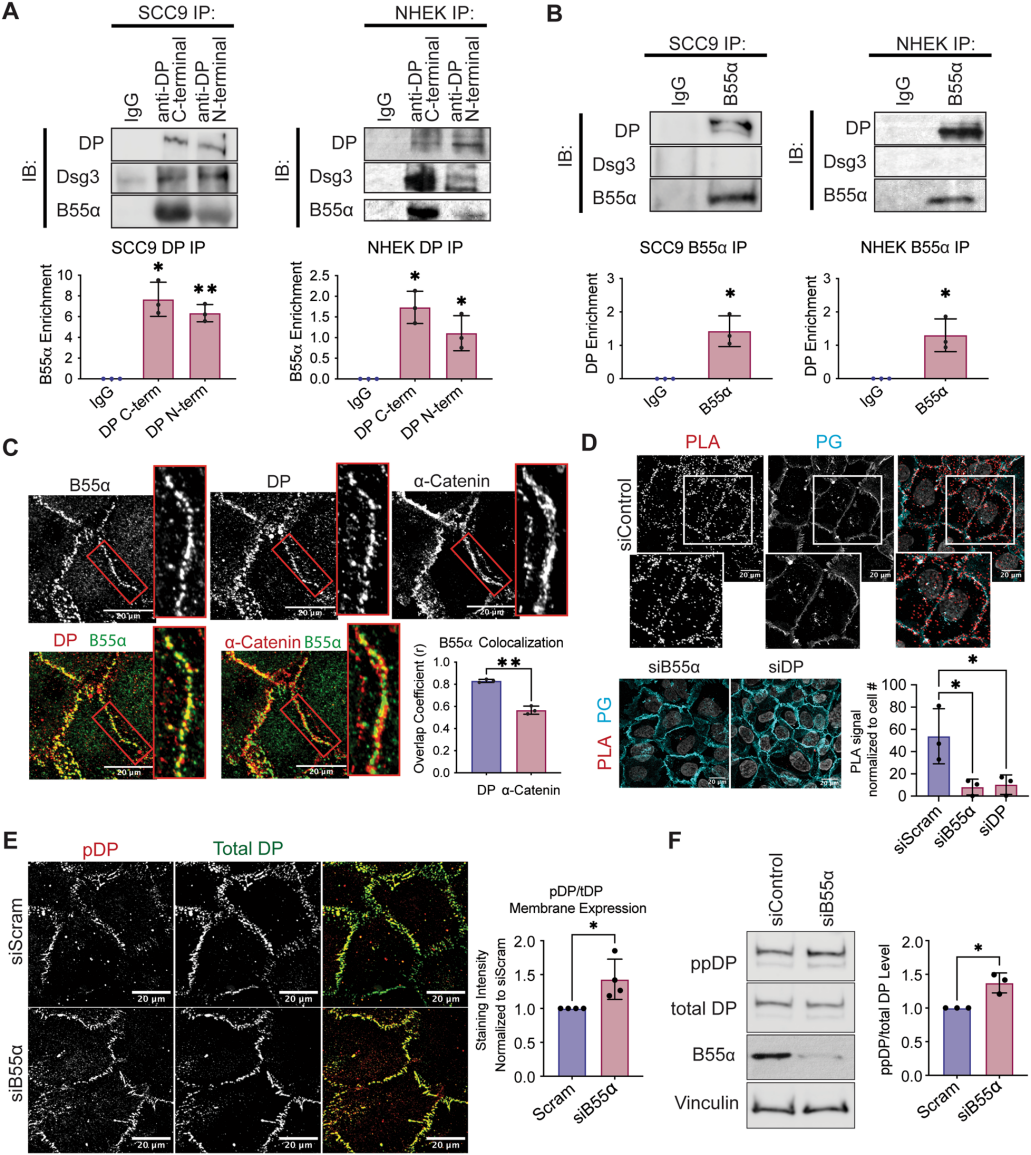
DP is found in complex with the PP2A regulatory subunit B55⍺ in SCC9 cells. (**A**) Immunoprecipitation of endogenous DP using antibodies targeting DP’s C-terminus or N-terminus and blotting back for Dsg3 or B55⍺. SCC9s (left) and NHEKs (right) were grown for 2 days in high-calcium media (HCM). (**B**) Immunoprecipitation of endogenous B55⍺ and blotting back for DP or Dsg3. SCC9s (left) and NHEKs (right) were grown for 2 days in high-calcium media (HCM). (**C**) SCC9 immunofluorescence co-stained for B55⍺, DP, and ⍺-Catenin. Overlayed images are shown below. Colocalization analysis as determined by an object-based colocalization analysis tool represented as overlap coefficient measurements. (**D**) Proximity ligation analysis performed on SCC9 cell transfected with siRNA targeting either a Scramble control, B55α, or DP. A fluorescence-based PLA signal was measured on fixed coverslips incubated with B55α and DP targeting antibodies. (**E**) Immunofluorescence staining of S2849 phosphorylated DP (pDP) in SCC9 cells transfected with siRNA targeting the B55α subunit. Staining intensity from was quantified at the membrane using PG stain as a mask. (**F**) Amount of the dual S2845/S2849 phosphorylated DP (ppDP) were analyzed in total SCC9 cells transfected with siRNA targeting the B55α subunit. Statistical analyses were performed using a One-way ANOVA with multiple comparisons (**A,D**) or a student t-test (**B**–**C**,**E**–**F**). * < 0.05; ** < 0.01; *** < 0.001.

### B55a exists in complex with Desmoplakin

PP2A is a heterotrimeric protein comprising a scaffolding subunit (A), a catalytic subunit (C), and a regulatory subunit (B). To form the holoenzyme, the A and C subunits bind one of 23 different B subunit isoforms which are responsible for binding the substrates^22^. To better understand how PP2A regulates DP, we sought to identify the B subunit responsible for targeting PP2A to DP. In an unbiased proteomics study, DP was identified as binding to the B subunit B55α^23^. Additionally, in both cell and mouse models, B55α loss resulted in impaired epidermal barrier development^24–27^. Based on these reports, we set out to address the extent to which B55α and DP associate in keratinocytes.

Toward this end, a co-immunoprecipitation (co-IP) assay was performed to pull down endogenous DP complexes using two different DP-directed antibodies targeting DP’s C- and N- termini in both SCC9 and NHEK cells. Both the C- and N-terminus targeting antibodies pulled down desmosomal cadherin Dsg3 as expected. In addition, B55α was also enriched in both IP complexes when compared to the IgG control (Fig. 2A). A co-IP using an antibody targeting endogenous B55α similarly resulted in an enrichment in DP compared to control, but interestingly did not enrich for Dsg3 suggesting B55α may preferentially interact with DP (Fig. 2B).

To look at potential B55α-DP co-localization, immunofluorescence was performed on SCC9 cells co-staining B55α with either DP or another membrane-associated protein including the adherens junction cytoskeletal linker protein α-catenin. B55α staining more closely resembled DP’s localization than α-Catenin (Fig. 2C). Co-localization was quantified using an object-based analysis tool, revealing significantly higher colocalization between B55α/DP than B55α/α-catenin^28^. Staining SCC9 cells transfected with B55α targeted siRNA confirmed that the membrane localized B55α signal is specific to B55α protein (Supplemental Fig. 3A). Notably, B55α loss was associated with a decrease in total amount of DP at the cell membrane suggesting B55α may regulate DP dynamics (Supplemental Fig. 3A). Additionally, loss of DP coincided with loss of membrane associated B55α, supporting the notion that B55α is physically anchored to DP at the membrane.

**Figure 3:**
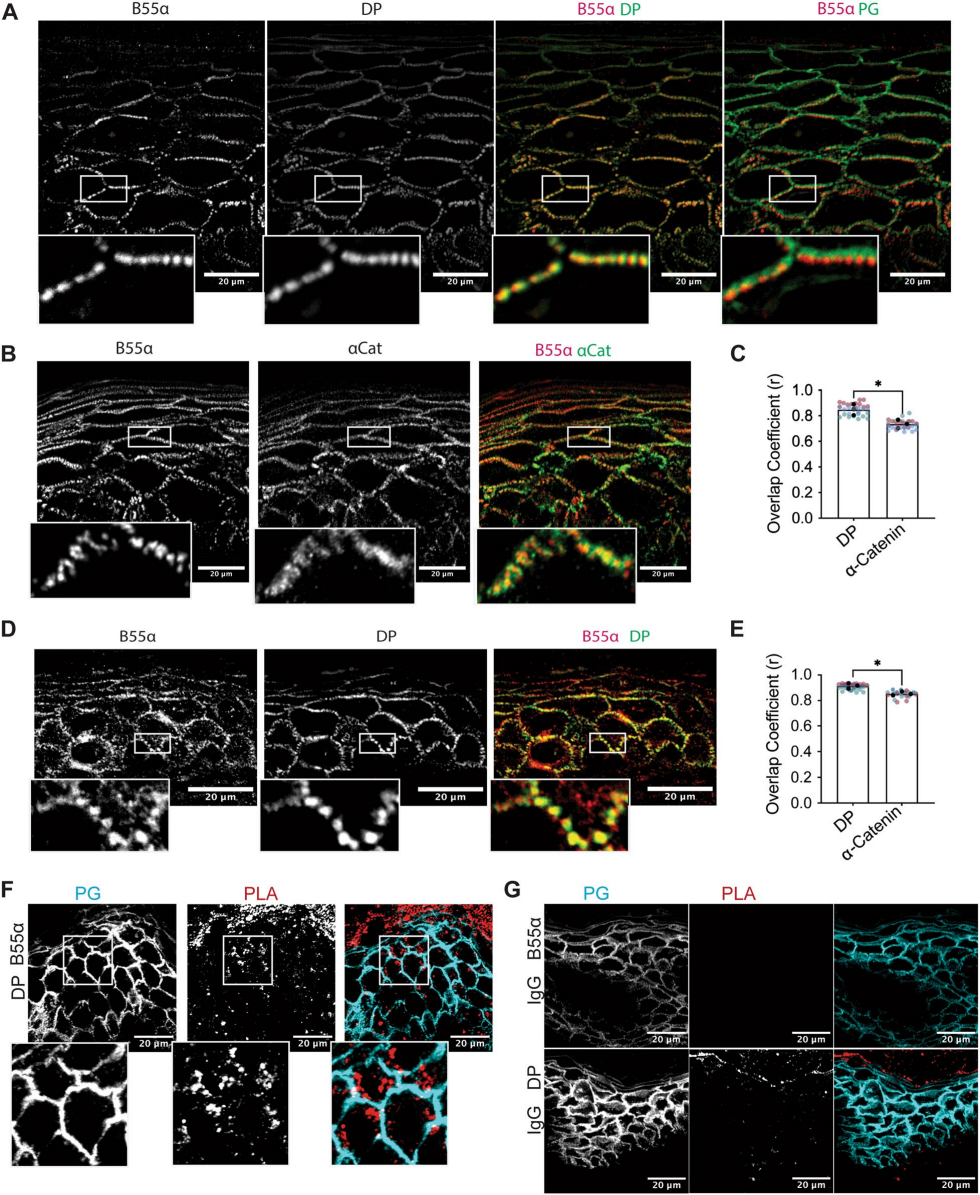
B55alpha is found localized to sites of intercellular membranes in the 3D epidermis in vitro and in vivo. (**A**–**B**) 3D organotypic reconstructed skin cultures grown for 6 days in culture and immunofluorescence staining of B55⍺, DP, and PG (**A**) and B55⍺, α-Catenin, and PG (**B**) was performed. (**C**) Colocalization analysis of (**A**–**B**) as determined by an object-based colocalization analysis tool represented as overlap coefficient measurements. (**D**) Human skin samples were stained for B55⍺ and DP using immunofluorescence. (**E**) Colocalization analysis of (**D**) and (Supplemental Fig. 4D) as determined by an object-based colocalization analysis tool represented as overlap coefficient measurements. (F–G) Proximity ligation analysis performed on fixed human skin samples. A fluorescence-based PLA signal was measure on fixed slides incubated with either B55α and DP targeting antibodies or B55α and IgG. Statistical analyses were performed using a student t-test. * < 0.05; ** < 0.01.

To validate this interaction in cells, a proximity ligation assay (PLA) using antibodies targeting B55α and DP was performed in SCC9s transfected with siRNA targeting either a Scramble control, B55α, or DP^29^. Consistent with the colocalization analysis, the PLA produced a significant signal in the Scramble control cells that was lost with the knockdown of either B55α or DP, confirming that the PLA signal is due to close proximity between the two proteins within cells (Fig. 2D, Supplemental Fig. 3B). To determine the functional importance of the DP-B55α complex, SCC9 cells were transfected with siRNA targeting B55α and were grown at confluency for 2 days. Immunofluorescence staining resulted in an increase in S2849 phosphorylated DP at cell membranes (Fig. 2E, Supplemental Fig. 3C). Additionally, western blot analysis confirmed that B55α knockdown increased the level of dual-phosphorylated DP in cells (Fig. 2F).

To determine if a B55α/DP complex also exists in tissues, a 3D organotypic reconstructed epidermal raft model grown for 6 days was co-stained for B55α and DP. Consistent with the cellular data, B55α was found localized at the membrane in a pattern more closely resembling DP than PG or α-Catenin (Fig. 3A–B, Supplemental Fig. 4A). Furthermore, object-based colocalization analysis revealed B55α had a higher overlap coefficient with DP than with either PG or α-Catenin (Fig. 3C, Supplemental Fig. 4B). Staining human skin samples also showed B55α preferentially colocalized with DP at the membrane within the intact epidermis suggesting this interaction is maintained in vivo (Fig. 3D–E, Supplemental Fig. 4C–D). To more directly interrogate the B55α-DP interaction in vivo, PLA was performed on human skin samples using B55α and DP directed probes and compared to samples treated with B55α antibody or IgG alone. Consistent with the cellular data, PLA signal was robustly detected compared to controls, further supporting the conclusion that B55α is in complex with DP within the intact stratified epidermis (Fig. 3F–G).

**Figure 4:**
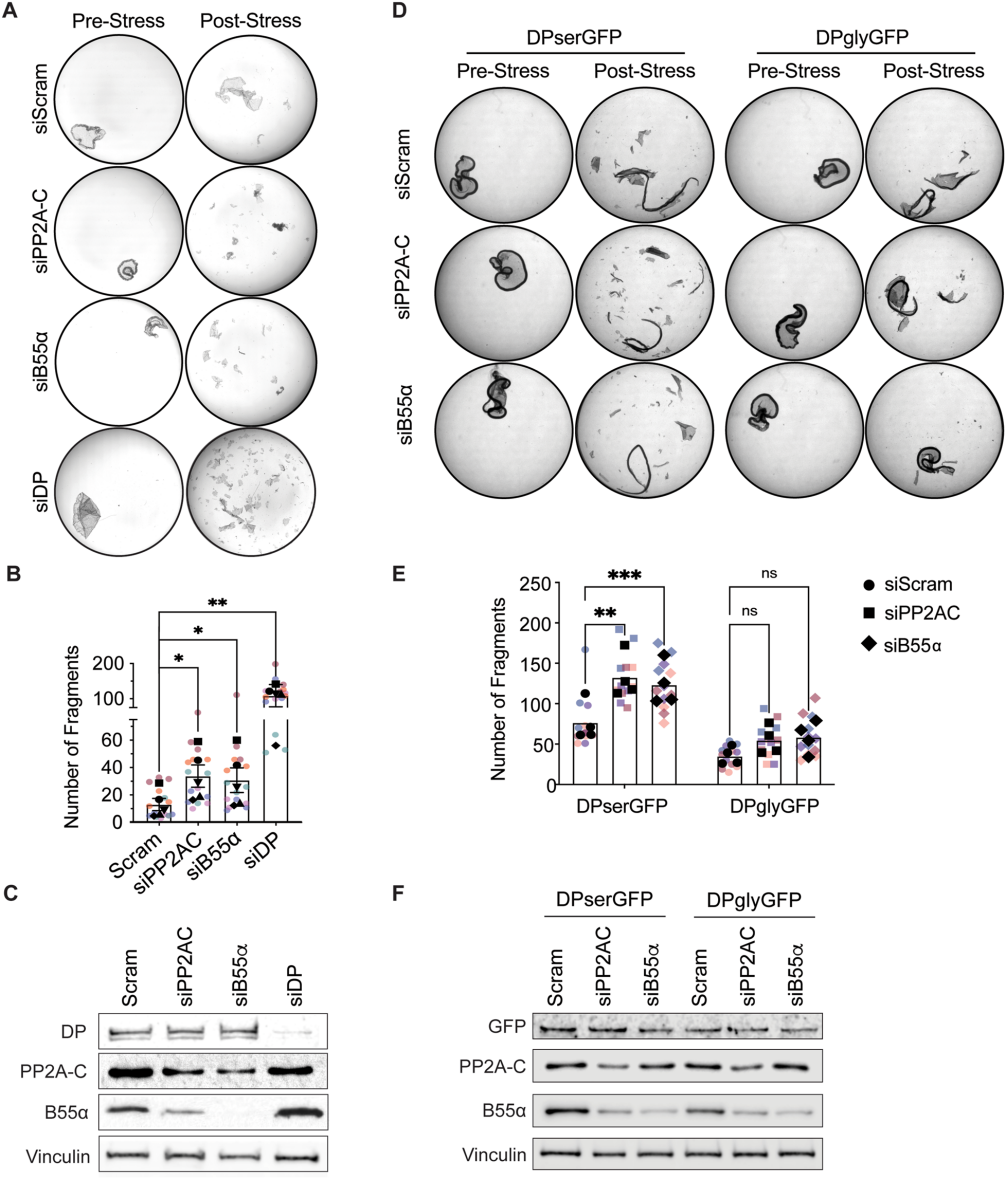
PP2A-B55alpha regulates epithelial cell adhesion and barrier through DP. (**A**) A dispase-adhesion assay was performed in SCC9s transfected with siRNA targeting Scramble, B55⍺, or DP and grown in normal HCM for 5 days. Images represent SCC9 monolayers pre- (left) and post- (right) exposure to mechanical stress. (**B**) Monolayer fragmentation from (**A**) was quantified and represented as total fragment number from n = 5. (**C**) Western blot validation of siRNA knockdown in (**A**). (**D**) A dispase-adhesion assay was performed in A431 cells expressing either dox-inducible DPserGFP or DPglyGFP and transfected with siRNA targeting Scramble, B55⍺, or DP. Cells were grown in normal HCM with 1 μg/mL Dox for 6 days. Images represent A431 monolayers pre- (left) and post- (right) exposure to mechanical stress. (**E**) Monolayer fragmentation from (**A**) was quantified and represented as total fragment number from n = 4. (**F**) Western blot validation of siRNA knockdown in (**C**). Statistical analyses were performed using a One-way ANOVA with multiple comparisons. * < 0.05; ** < .01.

### PP2A-B55α regulates desmosome-mediated cell adhesion

Our group previously reported that ectopic expression of constitutively hypo-phosphorylated DP C-terminus increases adhesion strength^17^. Therefore, we were interested in identifying the role of PP2A-B55α in regulating keratinocyte adhesion through DP’s phospho-regulation. A dispase-based adhesion assay was performed on SCC9 cells in which either the PP2A catalytic subunit (PP2A-C) or the B55α regulatory subunit was knocked down using siRNA. Cells were grown for 5 days in fully confluent monolayers and dispase treatment was used to lift cell monolayers off the culture plate before transporting monolayers into tubes to undergo mechanical perturbation through inversion. Adhesion strength was determined by recording the level of fragmentation of the monolayers. Silencing of either PP2A-C or B55α resulted in significantly more monolayer fragments when compared to controls (Fig. 4A–C). Notably, siDP treated cells resulted in complete fragmentation of cell monolayers suggesting PP2A-B55α loss only partially disrupts DP function.

To determine the extent to which effects of PP2A-B55α loss on adhesion were due to its phospho-regulation of the DP C-terminus, we utilized A431 cells expressing either a dox-inducible DP-GFP construct expressing wild-type DP (DPserGFP) or a previously described constitutively hypo-phosphorylated S2849G DP mutant (DPglyGFP)^17^. The A431 cells were transfected with siRNA targeting either a Scramble control, PP2A-C, or B55α. Following transfection cells were grown at confluency in media including 1 μg/mL dox for 6 days before monolayers were collected using dispase treatment and subjected to mechanical rotation. Consistent with the SCC9 data, PP2A-C and B55α knockdown increased fragmentation in DPserGFP expressing cells (Fig. 4D–F). Additionally, consistent with previously reports, DPglyGFP expression resulted in less fragmentation in Scramble control cells compared to DPserGFP expression. However, PP2A-C and B55α knockdown had no effect on fragmentation in the DPglyGFP expressing cells, suggesting the ability of PP2A-B55α to regulate keratinocyte adhesion is dependent on the phospho-regulation of DP’s C-terminus. Collectively, these studies present PP2A-B55α as a molecular module capable of modifying the adhesive strength of keratinocytes in response to their changing mechanical environments.

## Discussion

**Figure 5:**
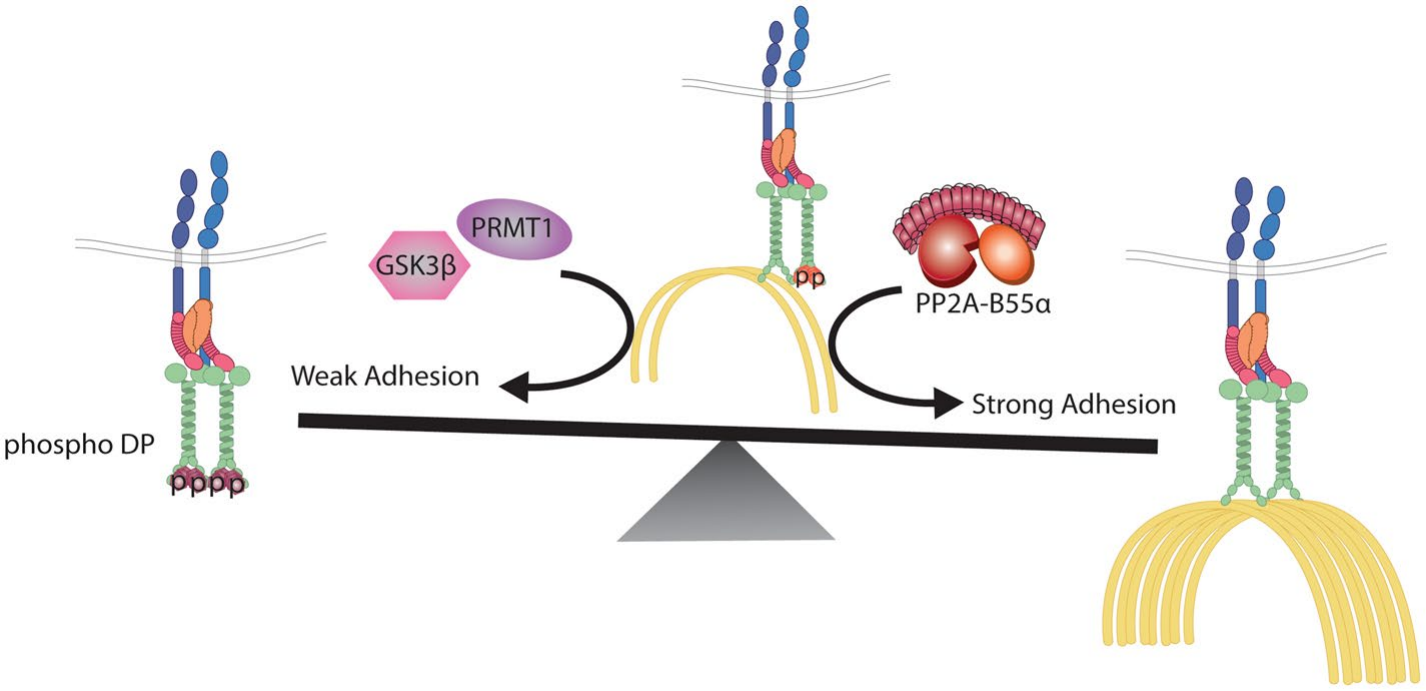
A balance between PP2A-B55alpha and GSK3β/PRMT1 activities on DP’s C-terminus controls desmosome-adhesion strength through regulating the DP-IF connection. A schematic representing the regulatory switch controlling desmosome-mediated adhesion through the phospho-regulation of the DP-IF interaction. Albrecht et al. previously identified GSK3β and PRMT1 as cooperating to induce the phosphorylation of DP’s C-terminus that inhibits DP-IF binding and weakens desmosomal adhesion. Our work has uncovered of PP2A-B55α as an opposing regulatory node capable of dephosphorylating DP’s C-terminus, inducing increased cell adhesion potentially through in increased association between DP and IF.

By identifying the phosphatase that acts on the phospho-motif at the DP C-terminus we have filled a major gap in our understanding of how the desmosome-intermediate filament connection is tuned through regulation of DP phosphorylation state. Our work shows that the PP2A-B55α holoenzyme is associated with the desmosomal cytoskeletal linker DP and loss of its activity results in an increase in total single and doubly phosphorylated DP as well as accumulation of hyper-phosphorylated DP concentrated at cell–cell junctions. We showed further that DP phosphorylation induced by inhibition of PP2A-B55α weakens intercellular adhesion, presumably through a decrease in DP-IF connection strength. The importance of the DP C-terminal GSR motif as a target for PP2A-B55α regulation is supported the fact that the DP S2849G mutant protects against the observed decrease in adhesion due to genetic depletion of B55α or the PP2A catalytic subunit. We previously demonstrated that the enzymes GSK3β and PRMT-1 cooperate to mediate DP phosphorylation^14^. Together with the current work, we propose an updated regulatory mechanism whereby the PP2A-B55α and PRMT-1/GSK3β form a molecular switch through the phospho-regulation of DP’s C-terminus to fine-tune the DP-IF connection that controls cell adhesive and tensile strength (Fig. 5).

PP2A-B55α activity has been associated with regulating several cellular processes including cell growth, DNA replication, mitotic exit, cytokinesis, and microtubule integrity^30,31^. Its most well characterized substrates include the tubulin binding protein tau, the spindle regulatory protein PRC1, the pocket protein p107, and many of the CDK1 cell cycle substrates. With the strong overlap of B55α substrates and cell cycle regulation, PP2A-B55α has primarily been thought of as a cell cycle regulatory enzyme. Our work presents a potential role for the cell cycle regulator PP2A-B55α in non-replicating differentiated keratinocytes that experience high levels of intercellular tension requiring a strong desmosome-IF anchor and no longer require high levels of cell cycle regulation.

Recent work in cardiomyocytes and simple epithelia revealed desmosomes to be mechano-sensitive structures, which respond to mechanical stress by recruiting desmosome proteins to sites of cell–cell adhesion^32,33^. Despite these reports, it is unclear how the desmosome may differentially impact epidermal mechanics across the stratified epidermis. One potential mechanism could be through its cytoskeletal connections. Consistent with this idea, interference with the DP-IF interaction inhibits tight junction formation in 3D models, supporting its importance for epidermal barrier function^34,35^. Given our previous observation that a hypo-phosphomimic of DP increases epithelial sheet stiffness^19^, our data raise the possibility that tuning DP-IF interactions through PP2A-B55α mediated de-phosphorylation could contribute to the stiffness gradient recently reported to exist in the stratified epidermis^36^. In turn, increased tension in the superficial epidermis would help maintain tight junctions in the superficial stratum granulosum 2 (SG2). This is consistent with previous studies showing PP2A catalytic activity and the B55α regulatory subunit specifically are required for normal epidermal development, barrier function and specifically tight junction formation in cellular and in vivo models of PP2A loss^24–27^. Furthermore, PP2A activation through topical plant extract treatments improved the epidermal barrier function in human subjects^37^. Together, these data uncover a possible mechanism to explain how the PP2A-B55α holoenzyme regulates both the epidermal barrier and tissue mechanics.

The importance of the DP-IF association is highlighted by the severe cardio-cutaneous diseases associated with mutations in DP’s C-terminus domain that interfere with the IF binding site and phospho-regulatory motif^9,13^. These include the cardiocutaneous disease Carvajal syndrome, associated with a truncation mutation at DP’s C-terminus, and the desmosome-related disease AC^10,38,39^. AC is associated with mutations in several desmosome components including a point mutation within DP’s C-terminus that interferes with the proper regulation of the phospho-regulatory motif that we have identified as a target of PP2A-B55α. Furthermore, ectopic expression of a constitutively hypo-phosphorylated DP mutant was sufficient to restore IF distribution and preserved cell–cell adhesion in a desmosome deficiency disease model of pemphigus vulgaris^40^. Notably, only half of all AC cases have been linked to mutations in known desmosome components^39^. The identification of PP2A-B55α as a regulator of DP’s C-terminus phospho-motif raises the possibility that mutations in the PP2A-B55α complex could be a candidate driving desmosomal diseases in patients without an identified desmosome mutation. Finally, previously published collaborative work showing PP2A inhibition increased phosphorylation of a conserved sequence in the more ubiquitously expressed plakin family relative Plectin, raises the possibility that B55α could play a broader regulatory role for plakins in different tissue types including cardiac and skeletal muscle^41^.

In summary, we have identified PP2A-B55α as a newly recognized component of a molecular switch also containing PRMT-1/GSK3β. Through its ability to negatively regulate a critical phospho-motif on DP’s C-terminus we propose that PP2A-B55α tunes desmosome-mediated adhesion to provide desmosomes with dynamic properties necessary for the development and maintenance of the epidermal barrier.

## Material and methods

### Cell lines, culture conditions, and treatments

Human-derived oral squamous cell carcinoma SCC9 cells (a gift from J. Rheinwald, Harvard Medical School, Boston, MA) were cultured in DMEM/F12, 10% FBS, and 1% penicillin/streptomycin. Immortalized human keratinocyte HaCaT cells were cultured in DMEM, 10% FBS, and 1% penicillin/streptomycin. Cell lines were maintained at 37 °C in a humidified atmosphere of 5% CO_2_. Normal Human Epidermal Keratinocytes (NHEKs) are isolated from neonatal foreskin provided by the Northwestern University Skin Biology and Diseases Resource-based Center (SBDRC) as previously described ^42^. NHEKs were maintained in M154 (Thermo Fisher Scientific) growth media supplemented with 0.07 mM CaCl_2_, human keratinocyte growth supplement (HKGS; Thermo Fisher Scientific), gentamicin and amphotericin B. NHEKs were used to generate 3D reconstructed epidermal organotypic cultures as previous described ^42^.

For siRNA transfection, cells were plated and grown overnight to 60% confluency before transfection with Dharmafect (Thermo Fisher Scientific). Dharmacon ON-TARGET siRNA’s (Horizon Discovery) were used to target PP2A-C(J-003598–09) and B55α(J-004824–06). For drug treatment studies, cells were grown at confluency for 3 days before treatment with OA (50 nM or 100 nM), Tautomycin (250 nM), or DMSO for 3 h. Doxycycline (Sigma Aldrich) was used at 1 μg/mL concentrations. NHEKs were grown to confluency and incubated in growth media supplement with 1.2 mM CaCl_2_ for 2 days prior to drug treatment.

### Immunofluorescence and microscopy

Cells plated on glass coverslips were fixed either in anhydrous ice-cold methanol for 3 min on ice to visualize phospho S2849 DP/total DP staining or 4% paraformaldehyde (PFA) solution for 20 min at room temperature followed by anhydrous ice-cold methanol for 3 min on ice to visualize B55α. Cells were blocked with either 5% goat serum or 1% BSA and 1% donkey serum. All samples were mounted onto glass slides with ProLong Gold antifade reagent (Thermo Fisher Scientific). 3D reconstructed skin and human skin samples were stained from frozen embedded samples and fixed as described above. For PLA analysis, samples were fixed as described above and PLA was performed as described in Hegazy et. al^29^.

Apotome images were acquired using ZEN 2.3 software with an epifluorescence microscope system (Axio Imager Z2, Carl Zeiss) fitted with an X-Cite 120 LED Boost System, an Apotome.2 slide module, Axiocam 503 Mono digital camera, and a Plan-Apochromat 40x/1.4, Plan-Apochromat 63x/1.4 objective, Plan-Apochromat 100x/1.4 objective (Carl Zeiss). Images are processed using ImageJ software. Colocalization analysis was performed using the JaCoP ImageJ plugin^28^.

### Western blot analysis

Whole cell lysates were generated using Urea Sample Buffer (8 M urea, 1% SDS, 60 mM Tris, pH 6.8, 5% b-mercaptoethanol, 10% glycerol). Proteins were separated by SDS-PAGE electrophoresis and transferred to nitrocellulose membranes. 5% milk was used to block membranes and dilute primary and secondary antibodies. Immunoreactive proteins were visualized using chemiluminescence or LI-COR fluorescence secondary antibodies. Images of all uncropped blots are included in Supplemental Fig. 5.

Phosphate-affinity SDS-PAGE was performed using Phos-tag (Wako Pure Chemical Industries). The procedure was performed following the manufacturer’s protocol (http://www.phos-tag.com). 50 μM of phos-tag acrylamide peptide is added during the preparation of a 15% wt/vol polyacryl- amide gel. Gel electrophoresis was run at 15mAmps for 5.5 h and transferred overnight onto a PVDF membrane. Subsequent immunoblotting was performed as described above. Quantification of bands was performed using Image Studio software (LI-COR).

### Antibodies and reagents

The following primary antibodies were used: NW6 Rabbit anti-DP C-terminal^43^; NW161 Rabbit anti-DP N-Terminal^44^; 11-5F Mouse anti-DP C-terminal (Sigma, Gift from D. Garrod^45^); anti–phospho-S2849 DP^14,41^; anti–dual phospho-S2845 and S2849 DP raised against a synthetic peptide corresponding to 2843–2853 of human DP (21st Century Biochemicals)^14^; anti-Desmoglein 3 5G11 (Sigma-Aldrich); 1407 Chicken anti-PG (Aves Laboratories); 2G9 Mouse anti-B55α (Cell Signaling Technology); PA5-18512 Goat anti-α-Catenin (Thermo Fisher Scientific); PA5-17443 Rabbit anti-α-Catenin (Thermo Fisher Scientific); mouse anti-S-Tag (EMD Millipore); Mouse anti-GAPDH (Santa Cruz Biotechnology); 12G10 Mouse anti-α-tubulin (Developmental Hybridoma Studies Bank); JL-8 Mouse anti-GFP (Clontech). The following secondary antibodies were used: Goat anti-Mouse IgG HRP (Cell Signaling Technologies); Goat anti-Rabbit IgG HRP (Cell Signaling Technology); Goat anti-Mouse conjugated with Alexa Fluor-488 (Thermo Fisher Scientific); Goat anti-Mouse conjugated with Alexa Fluor-568 (Thermo Fisher Scientific); Goat anti-Chicken conjugated with Alexa Fluor-647 (Thermo Fisher Scientific); Donkey anti-Goat conjugated with Alexa Fluor-488 (Thermo Fisher Scientific); Donkey anti-Mouse conjugated with Alexa Fluor-568 (Thermo Fisher Scientific); Donkey anti-Rabbit conjugated with Alexa Fluor-647 (Thermo Fisher Scientific).

### Co-IP assay

Cells grown at confluency for 3 days were lysed in NP40 (10 mM Tris–HCl pH8, 100 mM NaCl, 0.2% NP40, 10% glycerol, with protease inhibitor tablet) buffer on ice for 30 min. Supernatant was collected by centrifugation and incubated overnight at 4 °C rotating with anti-DP antibody or anti-B55α antibody conjugated to agarose (Sant Cruz Biotechnology). Agarose conjugated Protein A/G was rotated for 1 h at 4 °C before centrifugation and washes in 1× NP40 buffer. Samples were resuspended in 3× Lamelli Buffer and boiled at 100 °C for 10 min before western blot analysis was performed.

### Dispase assay

Cell monolayers were grown at confluency for 5 days in triplicate in 12-well plates. Cells were washed in PBS and treated with 2.4 U/mL of dispase (Sigma Millipore) diluted in PBS containing Ca^2+^ for 30 min. Lifted monolayers were placed in 15 mL conical tubes containing 3 mL PBS and placed in a rack. The rack was inverted 5–10 times as described in (Hudson et al.)^46^. Resulting fragments were returned to 12-well dishes and imaged using a dissecting microscope (MZ6; Leica).

### Supplementary Information

Supplementary Figures.

## Data Availability

No large datasets were generated or analyzed during the current study. All data generated or analyzed during this study are included in this published article and its supplementary information files. The datasets used and/or analyzed during the current study are available from the corresponding author upon reasonable request.
