# Supplementary material for: PP2A-B55alpha controls keratinocyte adhesion through dephosphorylation of the Desmoplakin C-terminus
Source: Sci Rep. 2023 Aug 5;13:12720. doi: 10.1038/s41598-023-37874-8 (PMC10404246; doi:10.1038/s41598-023-37874-8)
Supplement: Supplementary file 1 — Supplementary Figures. [file 41598_2023_37874_MOESM1_ESM.pdf]

Supplemental Figure 1

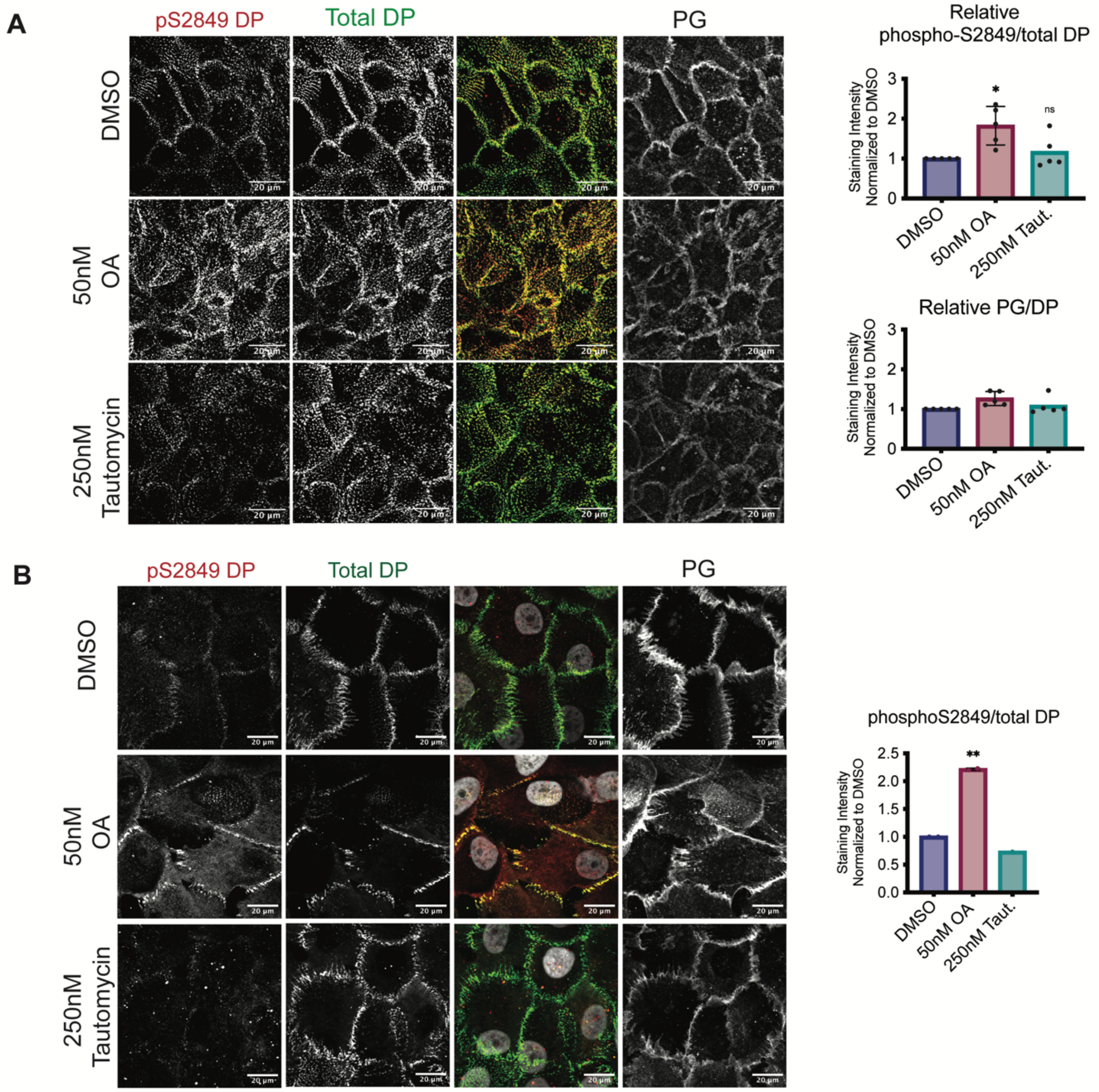

Supplemental Figure 1

HaCaTs [A] and NHEKs [B] were treated with either DMSO, 50nM OA or 250nM Tautomycin for 3 hours. Expression levels of S2849 phosphorylated (pDP) and total DP were analyzed by immunofluorescence staining. Staining intensity was quantified at the membrane using PG stain as a mask. Statistical analyses were performed on normalized data using a One sample t comparison to 1. * < 0.05; ** < .01.

Supplemental Figure 2

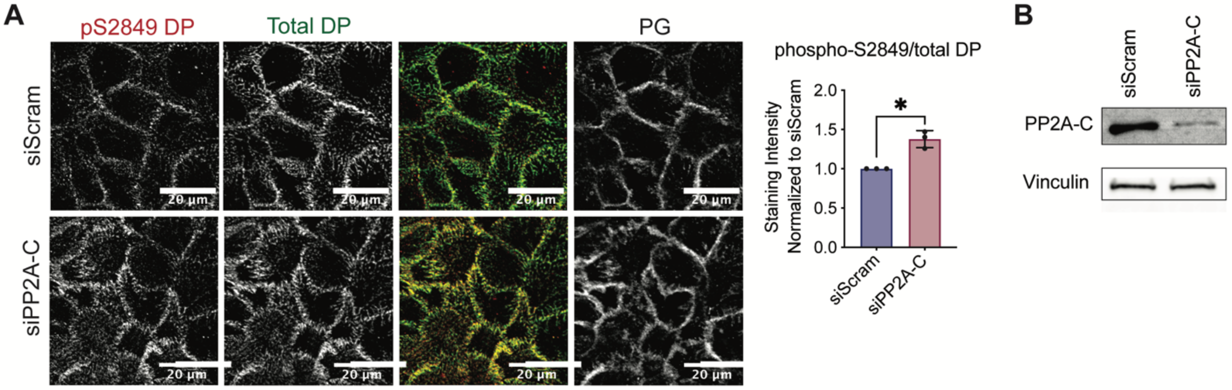

Supplemental Figure 2
[A] Immunofluorescence staining of S2849 phosphorylated DP (pDP) in HaCaT cells transfected with siRNA targeting the PP2A-C subunit. Staining intensity from was quantified at the membrane using PG stain as a mask. [B] Western blot validation of PP2A-C knockdown in cells from [A]. Statistical analyses were performed on normalized data using a student t-test. * < 0.05; ** < 0.01

Supplemental Figure 3

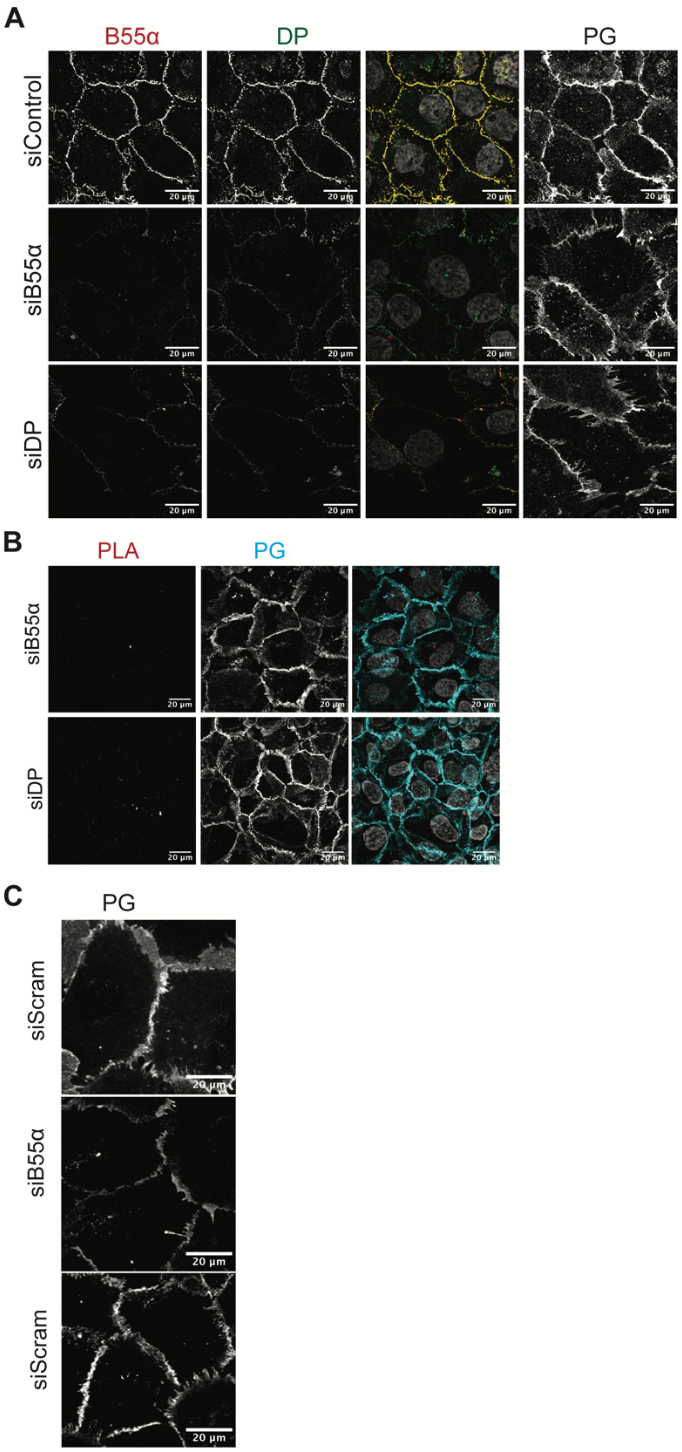

Supplemental Figure 3

Unmerged images of proximity ligation assay (PLA) in Figure 2E performed on SCC9 cells transfected with siRNA targeting either a scramble control, B55α, or DP. A fluorescence-based PLA signal was measure on fixed coverslips incubated with B55α and DP targeting antibodies. [B] SCC9s transfected with siRNA targeting B55α or DP and analyzed by immunofluorescence. [C] PG immunofluorescence staining of siB55□ transfected SCC9s from Figure 2E.

Supplemental Figure 4

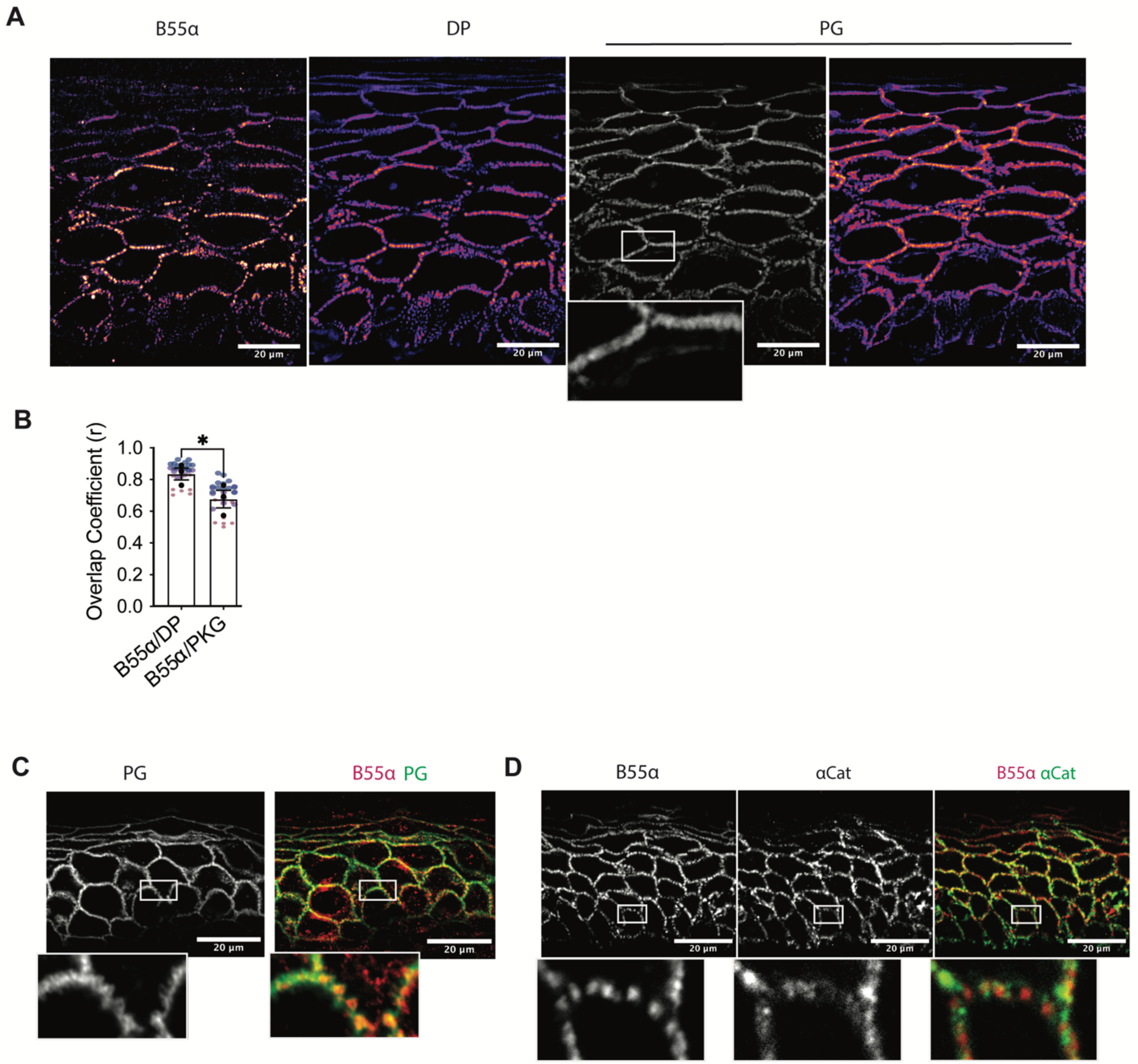

Supplemental Figure 4

[A] Staining intensities of B55α, DP, and PG across the 3D-reconstructed skin is shown using a HEAT map representation. [C] Colocalization analysis of Figure 3A as determined

by an object-based colocalization analysis tool represented as overlap coefficient measurements. [C-D] PG, B55α, and α-catenin staining in human skin samples.

Supplemental Figure 5: Uncut Blots

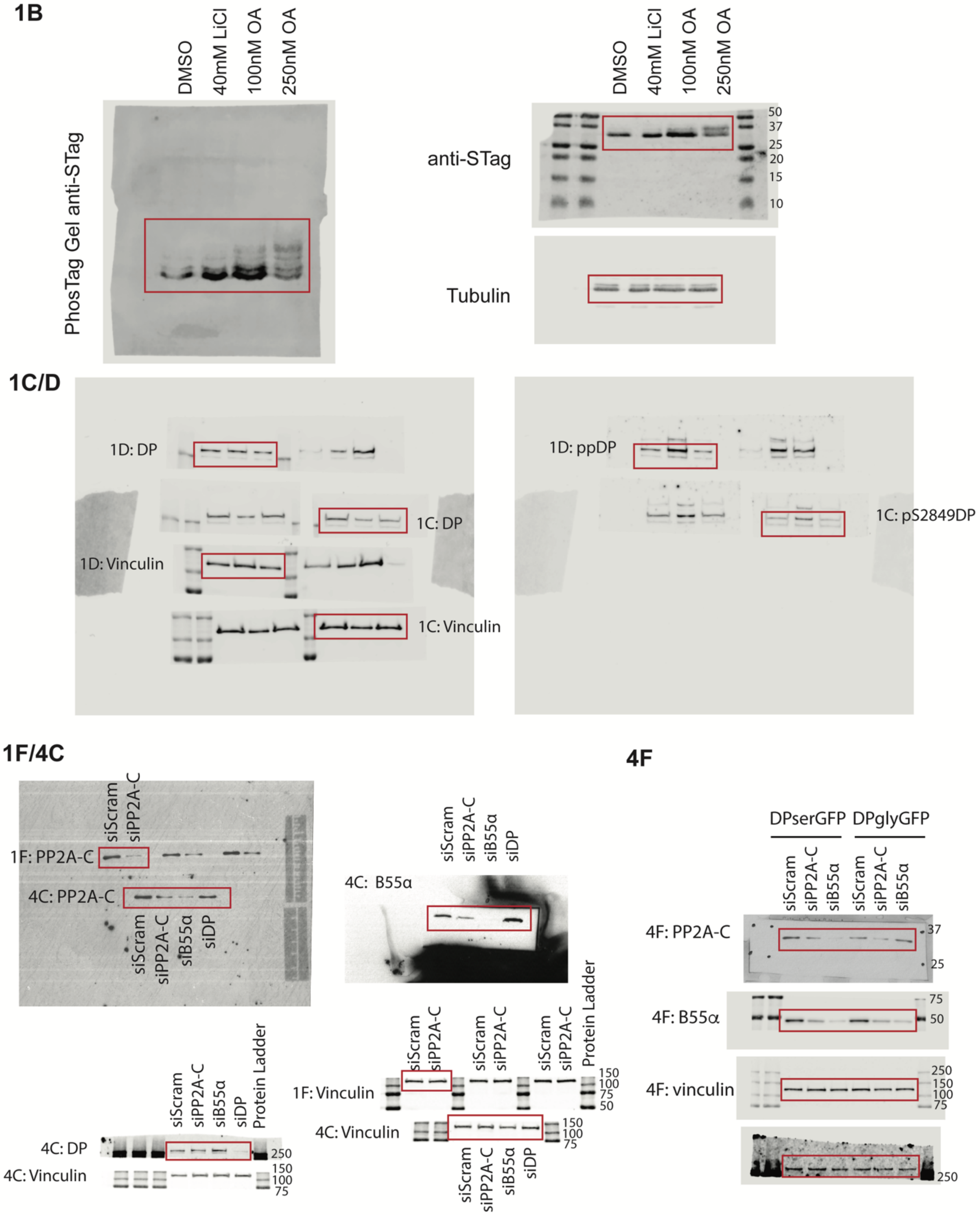

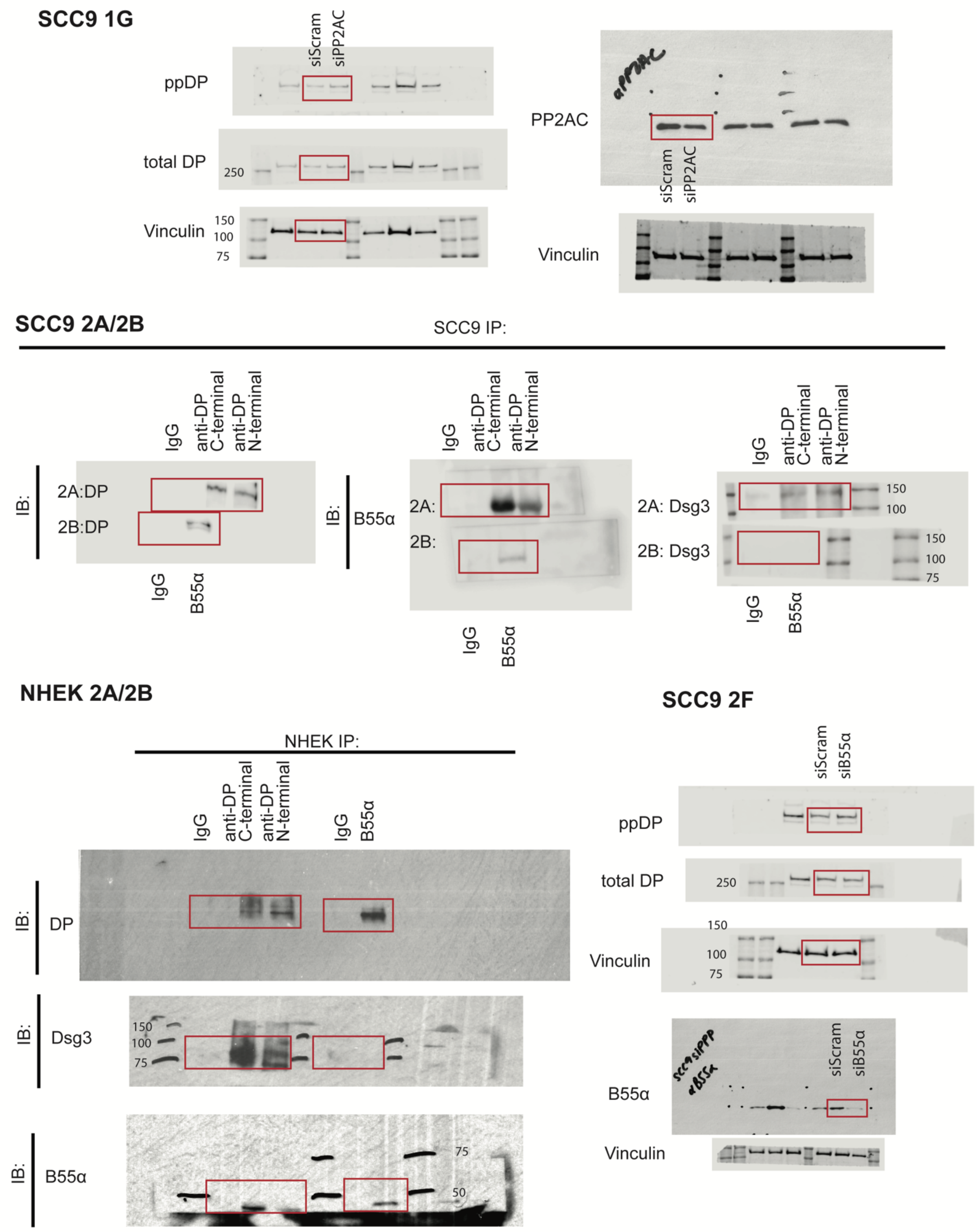

Supplemental Figure 5

All uncropped blots presented in manuscript are labeled and shown above.
